# Risk of Mental Health Problems in Children and Youths Following Concussion

**DOI:** 10.1001/jamanetworkopen.2022.1235

**Published:** 2022-03-07

**Authors:** Andrée-Anne Ledoux, Richard J. Webster, Anna E. Clarke, Deshayne B. Fell, Braden D. Knight, William Gardner, Paula Cloutier, Clare Gray, Meltem Tuna, Roger Zemek

**Affiliations:** 1 Children’s Hospital of Eastern Ontario Research Institute, Ottawa, Ontario, Canada; 2 Department of Cellular Molecular Medicine, University of Ottawa, Ottawa, Ontario, Canada; 3 ICES uOttawa, Ottawa, Ontario, Canada; 4 School of Epidemiology and Public Health, University of Ottawa, Ottawa, Ontario, Canada; 5 Department of Psychiatry, Children’s Hospital of Eastern, Ontario, University of Ottawa, Ottawa, Ontario, Canada; 6 Department of Pediatrics, Children’s Hospital of Eastern, Ontario, University of Ottawa, Ottawa, Ontario, Canada

## Abstract

**Importance:**

Concussion may exacerbate existing mental health issues. Little evidence exists on whether concussion is associated with the onset of new psychopathologies or long-term mental health problems.

**Objective:**

To investigate associations between concussion and risk of subsequent mental health issues, psychiatric hospitalizations, self-harm, or suicides.

**Design, Setting, and Participants:**

This population-based retrospective cohort study including children and youths aged 5 to 18 years with a concussion or orthopedic injury incurred between April 1, 2010, and March 31, 2020, in Ontario, Canada. Participants had no previous mental health visit in the year before the index event for cohort entry and no prior concussion or traumatic brain injury 5 years before the index visit. Data were collected from provincewide health administrative databases. Participants with concussion were included in the exposed cohort, and those with an orthopedic injury were included in the comparison cohort; these groups were matched 1:2, respectively, on age and sex.

**Exposures:**

Concussion or orthopedic injury.

**Main Outcomes and Measures:**

The primary outcome was mental health problems, such as psychopathologies and psychiatric disorders, identified from health care visits in emergency departments, hospitalizations, or primary care settings. Secondary outcomes were psychiatric hospitalizations, self-harm health care visits, and death by suicide (identified in health care or vital statistics databases).

**Results:**

A total of 152 321 children and youths with concussion (median [IQR] age, 13 [10-16] years; 86 423 [56.7%] male) and 296 482 children and youths with orthopedic injury (median [IQR] age, 13 [10-16] years; 171 563 [57.9%] male) were matched by age and sex. The incidence rates of any mental health problem were 11 141 per 100 000 person-years (exposed group) and 7960 per 100 000 person-years (unexposed group); with a difference of 3181 (95% CI, 3073-3291) per 100 000 person-years. The exposed group had an increased risk of developing a mental health issue (adjusted hazard ratio [aHR], 1.39; 95% CI, 1.37-1.40), self-harm (aHR, 1.49; 95% CI, 1.42-1.56), and psychiatric hospitalization (aHR, 1.47; 95% CI, 1.41-1.53) after a concussion. There was no statistically significant difference in death by suicide between exposed and unexposed groups (HR, 1.54; 95% CI, 0.90-2.61).

**Conclusions and Relevance:**

Among children and youths aged 5 to 18 years, concussion was associated with an increased risk of mental health issues, psychiatric hospitalization, and self-harm compared with children and youths with an orthopedic injury.

## Key Points

**Question:**

Is sustaining a concussion associated with increased risk of mental health problems among children and youths?

**Findings:**

This cohort study included 448 803 children and youths with concussion or orthopedic injury and found that children and youths who had sustained a concussion had a 40% increased risk of developing a mental health issue compared with age- and sex-matched children and youths with an orthopedic injury.

**Meaning:**

In this study, concussion was associated with an increased risk of mental health issues, psychiatric hospitalization, and self-harm among children and youths aged 5 to 18 years.

## Teaser

This cohort study investigates the associations between concussion and risk of subsequent mental health issues, psychiatric hospitalizations, self-harm, or suicides among pediatric patients.

## Introduction

Concussions are a serious concern in the pediatric population. In 2013, approximately 640 000 emergency department (ED) visits relating to pediatric traumatic brain injury (TBI; mostly concussion or mild TBI)^1,2^ were reported in the United States.^3,4^ From 2008 to 2013, rates of pediatric visits to EDs and primary care practitioners for concussion have increased by as much as 4-fold in the United States and Canada.^5,6,7^ Concussion symptoms last 2 to 4 weeks,^8^ but 30% of the pediatric population will experience persistent postconcussion symptoms—physical, emotional, cognitive, and sleep issues—beyond 1 month.^8,9^ The extent to which a concussion increases the risk of later psychopathology or new onset of psychiatric disorders is unclear.

A 2014 systematic review^10^ indicated a possible association between mental health problems (MHPs) and short-term recovery in youths who sustained a concussion. In most cases, these psychological, behavioral, and psychiatric problems seemed to resolve within 3 to 4 months.^10,11,12^ Some studies demonstrated possible associations between concussion and attention deficit/hyperactivity disorder,^13,14,15,16^ anxiety^17,18^ and depression symptoms,^19,20^ psychological distress,^21,22^ behavioral difficulties,^12,18^ psychiatric disorders,^23,24,25,26^ psychiatric visits and hospitalizations,^26^ suicidality,^27,28^ and suicide^28^; conversely, other analyses found no associations.^29,30,31,32^ Few studies have rigorously examined associations between concussion and risk of psychopathology, new onset of psychiatric disorders, or long-term MHPs.^32,33^ The studies that investigated these associations lacked adequate sample size^12,15,19,20,24^; had heterogeneous population samples, including more severe TBIs^13,15,18,19,20,23,24,32,33,34^; or had small or no comparison groups.^12,21,23,27^ Some studies did not adjust for important covariates, such as prior MHP,^13,19,23,27,32^ making it difficult to discern whether concussions precipitated new mental health symptoms or psychiatric disorders.^25^ Given that preexisting mental health is strongly associated with mental health post injury,^12^ the connections between concussion per se and the development of new and long-term MHP, self-harm, and suicide remain unclear. Research comparing a homogenous concussion sample with an appropriate comparison group while controlling for preinjury, injury, and case management factors is needed.

We conducted a population-based retrospective cohort study to investigate associations between concussion and incident psychopathologies in children and youths over a 10-year follow-up period. The primary objective was to investigate associations between concussion and MHPs. Secondary objectives were to examine associations between concussion and self-harm, psychiatric hospitalization, and suicide.

## Methods

### Study Design, Setting, and Participants

This retrospective cohort study examined children and youths aged 5 to 18 years who presented to an ED, primary health care, or mental health practitioner from April 1, 2010, to March 31, 2020, in Ontario, Canada. The project was approved by the Children’s Hospital of Eastern Ontario Research Ethics Board and the ICES Privacy Office and was reported according to the Strengthening the Reporting of Observational Studies in Epidemiology (STROBE) reporting guideline.^35^

We sequentially identified 2 cohorts: (1) children and youths with a concussion (exposed cohort) and (2) children and youths with an orthopedic injury (OI; comparison cohort). The patients in the exposed group were matched at a frequency of 1:2 to the comparison cohort on age and sex.^36^ OIs have been shown to be a good comparison group, as they share injury-related experiences (eg, ED visit, pain, injury-related stress) and premorbid characteristics.^37,38,39^ If a child or adolescent incurred more than 1 concussion or OI during the observational window, the first diagnosed concussion or OI visit to the ED or to a primary care practitioner was selected as the index event for cohort entry.

Children and youths in both cohorts were excluded if they did not have continuous Ontario Health Insurance Plan (OHIP; a universal health care system delivered to all Ontario residents at no direct cost) coverage during the 5 years before the index event; had a concussion or TBI during the 5 years before the index event; had an invalid death date (death date before or on the index date); were hospitalized on the day of the concussion or OI with a mental health outcome during the hospital stay; or had missing covariate data. To ensure that subjects were at risk for new mental health issues, we excluded children and youths with a health care visit with a mental health diagnosis in the year preceding the index event or a mental health diagnosis code during their index visit. If a child or adolescent sustained a concussion and an OI documented during the index visit, they were not excluded from the exposed group; because we identified the groups sequentially, those in the comparison group could not have had a concussion documented during any health care encounter.

### Follow-up Period

For both cohorts, follow-up began at the index event for cohort entry and continued until experiencing a study outcome, death, loss of OHIP eligibility, or March 31, 2020 (end of study period), whichever occurred first. Follow-up time ranged from 1 month to a maximum of 10 years. Because cause of death data were available only until the end of 2017, we restricted the follow-up period for suicide to December 31, 2017.

### Data Sources

Databases were linked using unique encoded identifiers and analyzed at ICES, a nonprofit research institute whose legal status under Ontario’s health information privacy law allows it to collect and analyze health care and demographic data, without consent, for health system evaluation and improvement. We used the Canadian Institute for Health Information (CIHI) National Ambulatory Care Reporting System (NACRS), which captures data from all ED visits. We used the OHIP database, which captures data on all primary care visits, to identify the cohorts (patients with concussion or OI). We used the CIHI Discharge Abstract Database (DAD), NACRS, OHIP and Ontario Mental Health Reporting System (OMHRS) to exclude patients with an MHP identified during the index visit or hospitalization. To identify those with a mental health outcome, self-harm visit, and specific health care conditions or comorbidities, we used the DAD, OHIP, OMHRS, and NACRS. The DAD and OMHRS were used to identify those with a psychiatric hospitalization visit. The Office of the Registrar General Data (ORGD) was used to identify death by suicide. The Registered Persons Database was used for sociodemographic characteristics. DAD, OHIP, OMHRS, and NACRS were used to identify patients’ health care conditions during the 5 years before the index event. All related codes are presented in eTable 1 in the Supplement.

### Exposure and Comparison Cohorts

The exposed cohort was identified using concussion-related ED visits (*International Statistical Classification of Diseases and Related Health Problems, Tenth Revision*, Canada [*ICD-10-CA*] diagnosis code of S060) or primary care visit (office, home, and long-term-care home visits with an OHIP diagnosis code of 850). Although concussion management has changed in the last decade, the concussion diagnostic definition remained constant throughout 2010 to 2020.^40,41,42^ The comparison cohort comprised children and youths with an OI, identified from diagnostic codes in NARCRS and OHIP (eTable 1 in the Supplement). We included only injuries managed with no open or closed reduction (surgical procedure) to eliminate the possibility of sedation or exposure to opioids, which has been associated with mental illnesses.^43^

### Outcomes

#### Primary Outcome

Our primary outcome was time to first diagnosis with a mental health condition during follow-up (eTable 1 in the Supplement). Mental health conditions were anxiety and neurotic disorders, adjustment reactions, behavioral disorders, mood and eating disorders, schizophrenia, substance use disorder, suicidal ideation, and disorders of psychological development. A previous validation study found Canadian health administrative data to have excellent specificity (97.0%-99.5%) and adequate sensitivity (22.3%-80.7%) for mental health service utilization.^44^

#### Secondary Outcomes

Three secondary outcomes were assessed: self-harm, psychiatric hospitalization, and death by suicide. Self-harm was defined as an ED visit or hospitalization with a self-injury or self-poison code (eTable 1 in the Supplement). Psychiatric hospitalization was defined as a hospitalization or OMHRS record with any of the *ICD-10-CA* codes for MHP or self-harm (eTable 1 in the Supplement). Death by suicide was determined with an ICES-validated method^45^ using *ICD-10-CA* codes X60-84 from the DAD and/or the ORGD.

### Demographic Variables and Covariates

Demographic variables and covariates were sex; age at index visit for cohort entry; neighborhood residential income quintile; and history of child abuse or neglect, migraines, organic mental disorders, developmental disorders, and pediatric complex chronic condition, identified within 5 years leading up to and the year of the index visit (eTable 1 in the Supplement).

### Statistical Analysis

Using a greedy matching algorithm, we matched the exposed and comparison cohorts at a 1:2 ratio on age and sex.^36^ A match was successful if age (completed years) and sex were exactly matched between patients with concussions and OI comparators.

Cox regression models were used to compute hazard ratios (HRs) and 95% CIs comparing incidence of outcomes in the exposed vs the comparison cohorts for each study outcome. Because socioeconomic status^46,47,48^ (measured indirectly by neighborhood income quintile^49^), child abuse and/or neglect,^50^ migraine, organic mental disorders, developmental disorders,^51^ and pediatric complex chronic conditions (eTable 1 in the Supplement),^52,53,54^ are associated with either the natural recovery of concussion or mental health, our model adjusted for these covariates. Clinical management of concussion changed over the study period; therefore, we adjusted for year of index visit to account for potential temporal confounding.^40,41,42^ The proportional hazards assumption was inspected for all models by plotting the log(−log[survival]) vs the log of survival and by including an interaction term between time and exposure group (concussion or OI) in the model.

Because the risk of subsequent concussions may be high^55^ and may increase the risk of mental health issues, we conducted a sensitivity analysis to assess whether number of concussions occurring after the index concussion would affect the main results. We introduced a time-dependent variable representing concussions during the follow-up period; any emergency or primary care practitioner visit resulting in a concussion diagnosis at 4 weeks or more after the index event for cohort entry was defined as a new concussion. This analysis also informs us on whether multiple concussions had a cumulative effect on the risk of mental health events.

Where applicable, a 2-sided *P* < .05 was considered statistically significant. All analyses were conducted using SAS Enterprise Guide version 7.1 (SAS Institute Inc).

## Results

**Figure 1.**
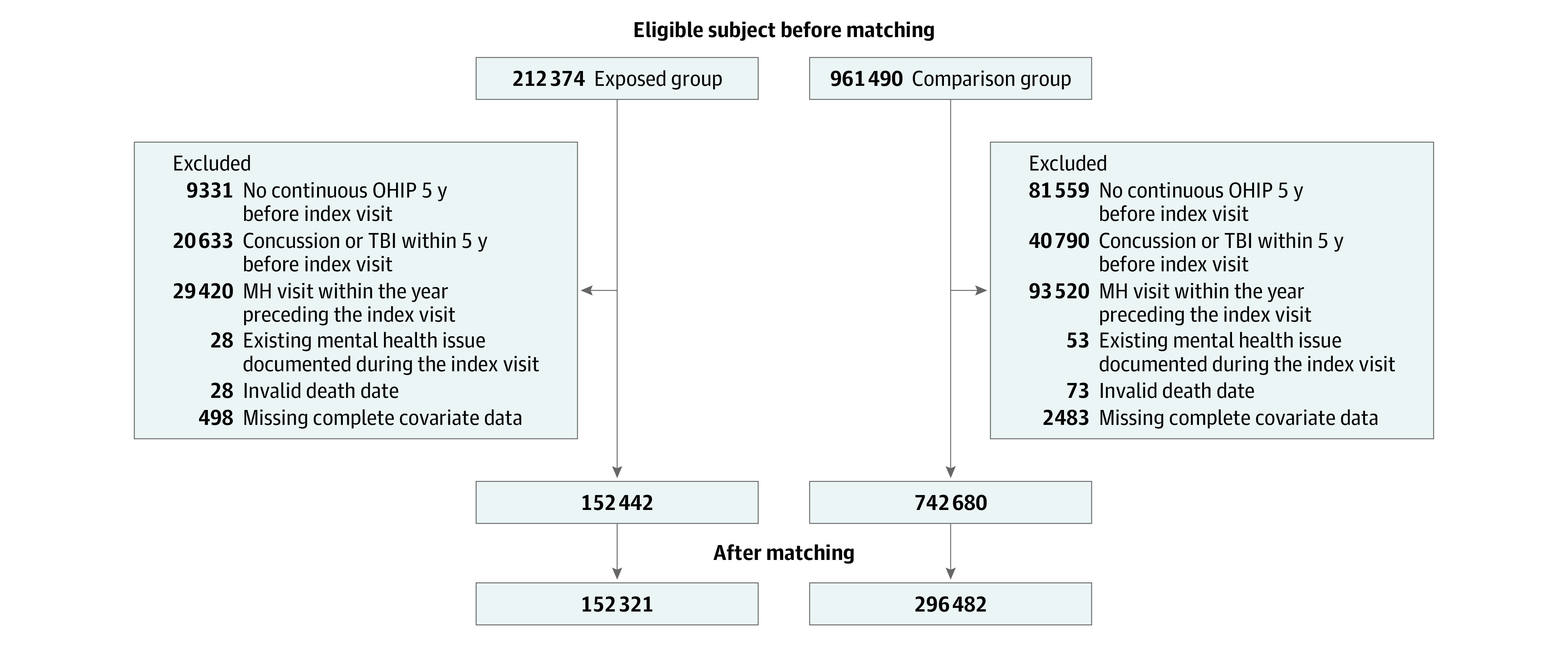
Study Flow Diagram MH indicates mental health; OHIP, Ontario Health Insurance Plan; and TBI, traumatic brain injury.

A total of 212 374 children and youths sustained 1 or more concussions in the study period (April 2010 to March 2020). After we applied exclusion criteria, 152 442 pediatric patients (median [IQR] age, 13 [10-16] years; 86 425 [56.7%] male) remained in the unmatched exposed cohort (Figure 1). A total of 961 490 children and youths sustained an OI during the study period, 218 810 of whom were excluded (Figure 1), leaving 742 680 participants (median [IQR] age, 12 [9-15]; 390 615 [52.6%] male) in the unmatched comparison group. Members of the exposed unmatched group had slightly higher prevalence of migraine, child abuse and/or neglect, and organic mental disorders, and tended to live in higher-income neighborhoods, compared with unmatched comparison group. After matching on age and sex, 152 321 children and youths were in the exposed matched group (median [IQR] age, 13 [10-16] years; 86 426 [56.7%] male), and 296 482 were in the comparison matched group (median [IQR] age, 13 [10-16] years; 171 563 [57.9%] male). Baseline characteristics of the study population were well-balanced across groups, and all standardized differences were less than 0.1, except for the lowest and highest neighborhood income quintiles (Table 1).

**Table 1.** Baseline Characteristics of Study Population

Baseline characteristics	Unmatched sample			Matched sample		
	Participants, No. (%)		Standardized difference	Participants, No. (%)		Standardized difference
	Exposed cohort (n = 152 442)	Comparison cohort (n = 742 680)		Exposed cohort (n = 152 321)	Comparison cohort (n = 296 482)	
Age at cohort entry median (IQR), y	13 (10-16)	12 (9-15)	0.26	13 (10-16)	13 (10-16)	0.03
Sex						
Male	86 425 (56.7)	390 615 (52.6)	0.08	86 423 (56.7)	171 563 (57.9)	0.02
Female	66 017 (43.3)	352 065 (47.4)	0.08	65 898 (43.3)	124 919 (42.1)	0.02
Neighborhood income quintile						
1 (lowest)	17 412 (11.4)	117 936 (15.9)	0.13	17 397 (11.4)	47 174 (15.9)	0.13
2	22 745 (14.9)	129 571 (17.4)	0.07	22 732 (14.9)	51 959 (17.5)	0.07
3	29 072 (19.1)	153 022 (20.6)	0.04	29 055 (19.1)	60 764 (20.5)	0.04
4	37 327 (24.5)	169 612 (22.8)	0.04	37 287 (24.5)	67 126 (22.6)	0.04
5 (highest)	45 886 (30.1)	172 539 (23.2)	0.16	45 850 (30.1)	69 459 (23.4)	0.15
Medical history						
Migraine	7487 (4.9)	22 926 (3.1)	0.09	7476 (4.9)	9448 (3.2)	0.09
Child abuse or neglect	256 (0.2)	1035 (0.1)	0.01	255 (0.2)	420 (0.1)	0.01
Organic mental disorders	739 (0.5)	365 (0.0)	0.08	739 (0.5)	145 (0.0)	0.08
Organic mental disorders or developmental disorders	127 (0.1)	1047 (0.1)	0.02	127 (0.1)	340 (0.1)	0.01
Pediatric complex condition						
Neurologic and neuromuscular	166 (0.1)	989 (0.1)	0.01	166 (0.1)	401 (0.1)	0.01
Cardiovascular	232 (0.2)	1127 (0.2)	0	232 (0.2)	466 (0.2)	0
Respiratory	65 (0.0)	386 (0.1)	0	65 (0.0)	160 (0.1)	0.01
Kidney and urologic	67 (0.0)	455 (0.1)	0.01	67 (0.0)	176 (0.1)	0.01
Gastrointestinal	204 (0.1)	1131 (0.2)	0	204 (0.1)	475 (0.2)	0.01
Hematologic or immunologic	217 (0.1)	1366 (0.2)	0.01	216 (0.1)	502 (0.2)	0.01
Metabolic	65 (0.0)	510 (0.1)	0.01	65 (0.0)	197 (0.1)	0.01
Other congenital or genetic defect	119 (0.1)	887 (0.1)	0.01	119 (0.1)	340 (0.1)	0.01
Malignant neoplasm	144 (0.1)	930 (0.1)	0.01	144 (0.1)	381 (0.1)	0.01
Premature and neonatal	15 (0.0)	79 (0.0)	0	15 (0.0)	15 (0.0)	0.01
Technology dependence and transplantation	69 (0.0)	503 (0.1)	0.01	69 (0.0)	185 (0.1)	0.01
Any congenital disease	2059 (1.4)	10 751 (1.4)	0.01	2058 (1.4)	4124 (1.4)	0
Miscellaneous or not elsewhere classified	288 (0.2)	1454 (0.2)	0	288 (0.2)	545 (0.2)	0

### Mental Health Outcomes

**Figure 2.**
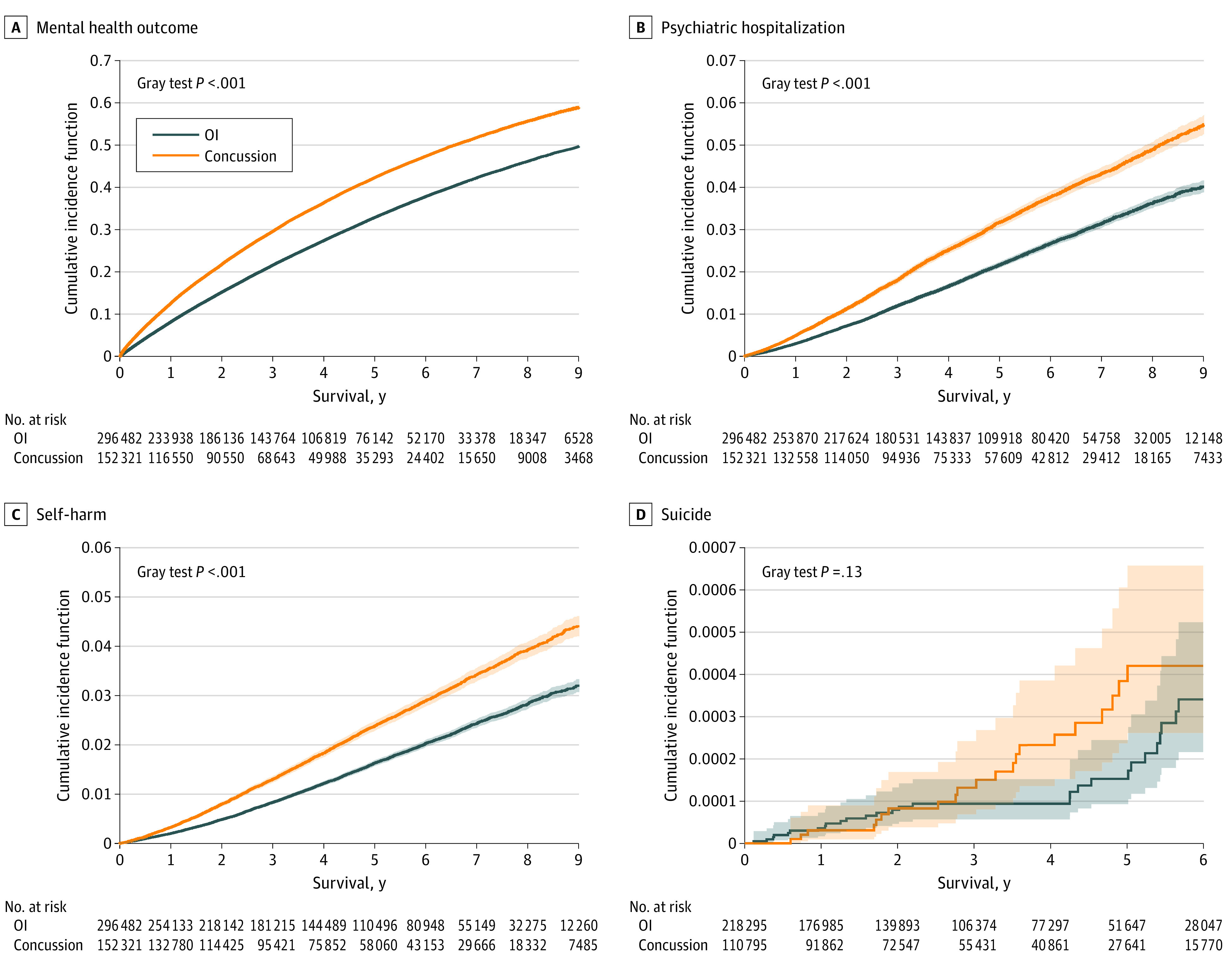
Cumulative Incidence of Mental Health Outcome, Psychiatric Hospitalization, Self-harm, and Suicide Shaded areas represent 95% CIs. OI indicates orthopedic injury.

A significant association was found between concussion and MHPs (incidence rates: exposed group, 11 141 [95% CI, 11 048-11 236] per 100 000 person-years; comparison group, 7960 [95% CI, 7905-8015] per 100 000 person-years; difference, 3181 [95% CI, 3073-3291] per 100 000 person-years; adjusted hazard ratio [aHR], 1.39; 95% CI, 1.37-1.40) (Table 2). Figure 2A depicts the higher cumulative incidence of MHP among children and youths with a concussion (*P* < .001). In addition to overall incidence, counts of anxiety and neurotic disorders, adjustment reactions and behavioral disorders, and mood disorders were higher in the exposed group than the comparison group (eTable 2 in the Supplement).

**Table 2.** Incidence Rates of MHPs, Self-harm, Psychiatric Hospitalization, and Suicide Outcomes

Outcome	Matched exposed group		Matched comparison group		Rate difference per 100 000 person-years (95% CI)	Hazard ratio (95% CI)	
	Events, No.	Incidence rate per 100 000 person-years (95% CI)	Events, No.	Incidence rate per 100 000 person-years (95% CI)		Crude^a^	Adjusted^b^
MHP	53 863	11 141 (11 048 to 11 236)	80 076	7960 (7905 to 8015)	3181 (3073 to 3291)	1.39 (1.37 to 1.40)	1.39 (1.37 to 1.40)
Self-harm	3072	475 (459 to 492)	4064	327 (317 to 337)	148 (128 to 168)	1.44 (1.37 to 1.51)	1.49 (1.42 to 1.56)
Psychiatric hospitalization	4013	623 (604 to 643)	5371	434 (442 to 446)	190 (167 to 212)	1.43 (1.37 to 1.49)	1.47 (1.41 to 1.53)
Death by suicide	24	7 (4 to 10)	30	4 (3 to 6)	2 (−1 to 5)	1.51 (0.88 to 2.58)	1.53 (0.90 to 2.61)

Abbreviation: MHP, mental health problem.

^a^ The matched sample was used to compute the crude hazard ratio.

^b^ Time to event analysis adjusting for residential neighborhood income quintile, child abuse or neglect, migraine history, organic mental disorders or developmental disorders, and pediatric complex chronic conditions. The rate differences were calculated prior to rounding.

### Self-harm and Psychiatric Hospitalization

A significant association emerged between concussion and self-harm (incidence rate: exposed group, 475 [95% CI, 459-492] per 100 000 person-years; comparison group: 327 [95% CI, 317-327] per 100 000 person-years; difference, 148 [95% CI, 128-168] per 100 000 person-years; aHR, 1.49 [95% CI, 1.42-1.56]) and psychiatric hospitalization (incidence rate: exposed group, 623 [95% CI, 604-643] per 100 000 person-years; comparison group, 434 [95% CI, 442-446] per 100 000 person-years; difference, 190 [95% CI, 167-212] per 100 000 person-years; aHR, 1.47 [95% CI, 1.41-1.53]). Outcome cumulative incidence curves are shown in Figure 2B and Figure 2C.

### Suicide

Of the 329 090 children and youths in the analysis of suicide (110 795 in exposed group; 218 295 in matched comparison group), 54 died by suicide in the 2010-to-2017 period (24 in matched exposed group; 30 in matched comparison group). Crude rates of suicide per 100 000 person-years were low and not statistically different between groups (exposed group, 7 [95% CI, 4-10] per 100 000 person-years; comparison group, 4 [95% CI, 3-6] per 100 000 person-years; aHR, 1.54 [95% CI, 0.90-2.62]) (Figure 2D).

### Sensitivity Analysis for Multiple Concussions

After introducing a time-varying variable representing subsequent concussions during follow-up, index concussion remained significantly associated with risk of mental illness (aHR, 1.34; 95% CI, 1.33-1.36), psychiatric hospitalization (aHR, 1.42; 95% CI, 1.36-1.48), and self-harm (aHR, 1.44; 95% CI, 1.38-1.52) but not death by suicide (aHR, 1.46; 95% CI, 0.85-2.52) (eTable 3 in the Supplement). The magnitudes of these associations remained consistent with the main analyses. Associations were found between having concussions beyond the index visit and mental health issues (HR, 1.09; 95% CI, 1.08-1.10), psychiatric hospitalization (HR, 1.07; 95% CI, 1.06-1.08), and self-harm (HR, 1.06; 95% CI, 1.04-1.07) but not death by suicide (HR, 1.09; 95% CI, 0.89-1.32) (eTable 4 in the Supplement).

## Discussion

In this population-based retrospective cohort study, children and youths who sustained a concussion had a higher risk of subsequent MHP than those who had an OI. Children and youths with a concussion had a higher risk of being hospitalized for a psychiatric disorder as well as risk of self-harm.

Preconcussion mental health has been identified as strongly associated with postinjury MHPs.^12,21,25,42,43,56^ It is postulated that concussion exacerbates preinjury MHPs.^12^ According to a recent systematic review and meta-analysis, psychiatric history explained 38% to 65% of the variance in postconcussion MHPs.^12^ Some have suggested that concussion is also associated with the onset of new mental health issues and psychiatric disorders.^25,26^ To verify whether concussion is associated with new psychopathologies and psychiatric disorders, we excluded children and youths with mental health contact during the year before their index visit for cohort entry. Even with this exclusion, concussion remained moderately associated with mental health disorders and psychiatric hospitalization. Our results are similar to a population-based study conducted in Sweden between 1973 and 1985, which found that mild TBI occurring between ages of 0 to 25 years was associated with an elevated risk of psychiatric visits and hospitalization and disability pension during adulthood, despite a definition of concussion and clinical management protocols that differed from ours.^26^

Our study found that, compared with an OI group, rates of postconcussion mental health visits were high and were associated with new MHPs. In Ontario, 1 in 5 children and/or youths has a mental health disorder.^57^ The proportion of children and youths with an MHP was higher in both of our study groups. This may be due to differences in the time period and method of identifying and/or defining mental health problems. It may also reflect the mutual experience of trauma, as it has been reported that individuals with OI may have long-lasting behavioral changes after injury.^39,58^ Our findings suggest that during concussion follow-up visits, physicians should assess patients’ mental health. It has been found that collaborative care and mental health treatment improve outcomes in pediatric concussion with chronic symptoms.^59^ Future studies should examine acute management protocols and strategies for reducing the risks of later mental health disorders among patients with concussion.^16,60^

Consistent with smaller cohort studies,^27,28^ we found that concussions were associated with a significantly increased risk of self-harm. In contrast to other studies,^27,28^ we did not find a significantly higher risk of suicide. This was likely due to the low number of deaths by suicide in this population. Despite not being statistically significant, it is clinically relevant that the concussion group had approximately twice the incidence rate of suicide; thus, health care practitioners should monitor suicidality and self-harm behaviors in children and youths after concussion.

Youth who have incurred a concussion are at 3 times higher risk of sustaining a subsequent concussion.^55^ Multiple concussions may increase the likelihood of MHPs. However, even when concussions after the index concussion were taken into account, mental health disorders, psychiatric hospitalization, and self-harm remained significantly associated with concussion, and the magnitude of risk of developing one of these outcomes did not change.

This study has several strengths. This was a large population-based study conducted over 10 years for the entire province of Ontario (approximately 14 million residents). Given differences in concussion management and increased media attention and community awareness throughout 2010 to 2020, we adjusted for year of index injury. In both exposure groups, we used routinely collected data to exclude participants with previous mental health visits, making it possible to examine whether concussions were associated with new-onset of an MHP. We used a comparison group exposed to orthopedic trauma and matched participants on sex and age. Previous studies that used comparison groups of healthy noninjured youths may have failed to control for the nonspecific associations of sustaining an injury and related preexisting risk factors.^33^

### Limitations

This study has limitations, including the retrospective observational design, which has inherent weaknesses.^61^ Concussion, OI, and mental health outcomes were defined using diagnosis codes in health administrative databases, thereby introducing the possibility of exposure or outcome misclassification. Few validation studies have examined the specific mental health and clinical codes used in this study.^44^ However, previous population-based concussion studies using a similar approach found that the *ICD-10* code for concussion diagnosis had a high positive predictive value.^62^ Canadian administrative data for MHPs have shown excellent specificity and adequate sensitivity for mental health service utilization.^44^ We used mental health coded visits as a proxy for mental health diagnoses, but the databases may not have captured some mental health services (eg, psychologists). However, misclassification of our outcomes due to this issue would have been nondifferential by exposure group, resulting in underestimation of the magnitude of true associations. We could not adjust for potential confounding factors, such as family anxiety, the psychosocial consequences of postconcussion symptoms, coping skills, management protocol, and a sedentary lifestyle, that may increase MHP risk.^32,63^ Children and youths with a previous diagnosis of substance use disorder were excluded, but we were unable to exclude those who had medical exposure to opioids before the index visit, which may increase the risk of MHP.

## Conclusions

In this study, concussion was associated with an increased risk of mental health visits, psychiatric hospitalization, and self-harm among children and youths aged 5 to 18 years who had sustained a concussion compared with their contemporaries who had sustained an OI. Our results suggest that clinicians should (1) assess for preexisting and new mental health symptoms throughout concussion recovery; (2) treat mental health conditions or symptoms or refer the patient to a specialist in pediatric mental health; and (3) assess suicidal ideation and self-harm behaviors during evaluation and follow-up visits for concussion.

## Notes

Supplement. **eTable 1.** Diagnostic Codes for Groups, Outcomes, and Covariates **eTable 2.** Types of Mental Health Issues for the Matched Exposed and Comparison Groups **eTable 3.** Association Between Concussion and Outcome Measures When Adjusting for Multiple Concussions **eTable 4.** Association Between Multiple Concussions After the Index Visit and Study Outcome Measures
