# Supplementary material for: Risk of Mental Health Problems in Children and Youths Following Concussion
Source: JAMA Netw Open. 2022 Mar 7;5(3):e221235. doi: 10.1001/jamanetworkopen.2022.1235 (PMC8902648; doi:10.1001/jamanetworkopen.2022.1235)
Supplement: Supplement. — eTable 1. Diagnostic Codes for Groups, Outcomes, and Covariates eTable 2. Types of Mental Health Issues for the Matched Exposed and Comparison Groups eTable 3. Association Between Concussion and Outcome Measures When Adjusting for Multiple Concussions eTable 4. Association Between Multiple Concussions After the Index Visit and Study Outcome Measures [file jamanetwopen-e221235-s001.pdf]

Supplemental Online Content

Ledoux AA, Webster RJ, Clarke AE, et al. Risk of mental health problems in children and youths following concussion. *JAMA Netw Open.* 2022;5(3):e221235. doi:10.1001/jamanetworkopen.2022.1235

eTable 1. Diagnostic Codes for Groups, Outcomes, and Covariates

eTable 2. Types of Mental Health Issues for the Matched Exposed and Comparison Groups

eTable 3. Association Between Concussion and Outcome Measures When Adjusting for Multiple Concussions

eTable 4. Association Between Multiple Concussions After the Index Visit and Study Outcome Measures

This supplemental material has been provided by the authors to give readers additional information about their work.

© 2022 Ledoux AA et al. *JAMA Network Open.*

eTable 1. Diagnostic Codes for Groups, Outcomes, and Covariates				
	**Variable**	**NACRS ICD-10**	**OHIP**	**OMHRS**
**Exposure group**				
	Concussion	S060	850	NA
	**Orthopedic Injury**	S42000, S42010, S42020, S42090, S42200,S42210, S42220, S42280, S42290, S42300, S42390, S42480, S42490, S43400, S43401, S43402, S435, S52000, S52010, S52090, S52100, S52110, S52120, S52190, S52200, S52300, S52500, S52580, S52590, S52600, S52900, S530, S62000, S62200, S62210, S62220, S62290, S62300, S62310, S62320, S62500, S62510, S62511, S62570, S62571, S62590, S62591, S62600, S62610, S62690, S62800, S63100, S63110, S63180, S63190, S6350, S6351, S6358, S6359, S6360, S6361, S6368, S6369, S6370, S6371, S6372, S6378, S6379, S698, S699, S82000, S82500, S82600, S82890, S8320, S8321, S8329, S833, S83401, S83411, S83481, S83491, S83501, S83511, S83581, S83591, S836, S92300, S92400, S92500, S92900, S93100, S93110, S9340, S9341, S9342, S9348, S9349, S9350, S9351, S936	810, 812, 813, 814, 815, 824, 834, 840, 842, 844, 845	
	Traumatic brain or head injury	S061, S062, S063, S064, S065, S066, S068, S069, S070, S071, S078. S079, S080, S088.	854	NA
**Outcomes**	**Mental health disorders**	**NACRS and DAD**	***OHIP***	***OMHRS***
	Anxiety and neurotic disorders	F40 F41 F42 F44 F45 F48 F930 F931 F932	300	B1O B1P B1Q B1R B1S B1T B1R Q1G_RETIRED_2016 Q1H_RETIRED_2016 L4J<br>Q1E Q1I
	Adjustment Reaction	F43	309	NA

© 2022 Ledoux AA et al. *JAMA Network Open.*

	Behavioral disorders	F90 F91 F92 F933 F938 F939 F94 F95 F98 F60 F61 F62 F63 F64 F65 F66 F68 F69 F21	307 313 314 301 302	E1A E1B E1C E1D E1E E1F E1G I2A I2B I2C I2D I2E I2F I2G Q1R Q1P_RETIRED_2016
	Mood	F31 F30 F32 F33 F34 F38 F39	296 311	B1H B1I B1J B1K B1L B1M B1N Q1F_RETIRED_2016
	Eating disorders	F50	NA	L4H N3A N3B N3C Q1L_RETIRED_2016 Q1J
	Other HH	F99	NA	NA
	Disorders of psychological development	F8	299 315 330	E2
	Schizophrenia	F20, F22, F23, F24, F25, F28, F29	295 297 298	Q1E_RETIRED_2016 Q1B
	Substance use disorder	F10 F11 F12 F13 F14 F15 F16 F17 F18 F19	291 303 292 304 305	C1 C2E C2C C2D C2B C2F Q1D_RETIRED_2016 C2A Q1P
	Suicidal ideation	R45.8	-	-
	**Self harm**			
	Self-Injury including undetermined intent	X6 X7 X80 X81 X82 X83 X84 Y10 Y11 Y12 Y13 Y14 Y15 Y16 Y17 Y18 Y19 Y20 Y21 Y22 Y23 Y24 Y25 Y26 Y27 Y28 Y29 Y30 Y31 Y32 Y33 Y34	NA	D1A D1A_RETIRED_2009 D1B D1B_RETIRED_2009 D1C D1C_RETIRED_2009 D1DA D1DB
	**Suicide**	X60-X84[40] captured in ORGD until the end of 2017<br>Or where manner_of_death=4 from ORGD		
**Covariates**		**NACRS and DAD**	***OHIP***	***OMHRS***

© 2022 Ledoux AA et al. *JAMA Network Open.*

	Child Abuse or Neglect	F51 F52 F53 F54 F55 F59	306	NA
	Migraine: Migraine	G43	346	NA
	Organic mental disorders, or developmental disorders	F70 F71 F72 F73 F78 F79	319	
	Organic: Organic mental disorders	F00 F01 F02 F03 F05 F06 F07 F09	290	
	Pediatric complex chronic condition:<br>Neurologic and neuromuscular, Cardiovascular, Respiratory, Renal and urologic, Gastrointestinal, Hematologic or immunologic, Metabolic, Other congenital or genetic defect, Malignancy, Premature and neonatal, Technology dependence and transplantation, Any congenital disease	The pediatric complex chronic condition codes were derived from Feudtner et al., 2014[41] study and procedure codes and the technology dependence and transplantation code was derived from the Cohen et al., 2012.[42]		

© 2022 Ledoux AA et al. *JAMA Network Open.*

eTable 2. Types of Mental Health Issues for the Matched Exposed and Comparison Groups

Variable	Exposed N(%) N=152,321	Comparison N(%) N=296,482	standard_ difference
≥ 1 visit anxiety & neurotic disorders			
No	114,074 (74.9%)	240,958 (81.3%)	0.15
Yes	38,247 (25.1%)	55,524 (18.7%)	
≥ 1 visit adjustment reaction			
No	145,661 (95.6%)	287,777 (97.1%)	0.08
Yes	6,660 (4.4%)	8,705 (2.9%)	
≥ 1 visit behavioral disorders			
No	131,093 (86.1%)	267,620 (90.3%)	0.13
Yes	21,228 (13.9%)	28,862 (9.7%)	
≥ 1 visit mood disorders			
No	138,642 (91.0%)	277,435 (93.6%)	0.1
Yes	13,679 (9.0%)	19,047 (6.4%)	
≥ 1 visit eating disorders			
No	151,859 (99.7%)	295,929 (99.8%)	0.02
Yes	462 (0.3%)	553 (0.2%)	
≥ 1 visit other mental health			
No	152,259 (100.0%)	296,400 (100.0%)	0.01
Yes	62 (0.0%)	82 (0.0%)	
≥ 1 visit disorders of psychological development			
No	150,116 (98.6%)	292,683 (98.7%)	0.01
Yes	2,205 (1.4%)	3,799 (1.3%)	
≥ 1 visit: Schizophrenia			
No	151,263 (99.3%)	294,777 (99.4%)	0.02
Yes	1,058 (0.7%)	1,705 (0.6%)	
≥ 1 visit: substance use disorder			
No	145,892 (95.8%)	287,134 (96.8%)	0.06
Yes	6,429 (4.2%)	9,348 (3.2%)	
≥ 1 visit suicidal ideation			
No	149,173 (97.9%)	292,363 (98.6%)	0.05
Yes	3,148 (2.1%)	4,119 (1.4%)	
≥ 1 visit: any mental health outcome			
No	98,458 (64.6%)	216,406 (73.0%)	0.18
Yes	53,863 (35.4%)	80,076 (27.0%)	

© 2022 Ledoux AA et al. *JAMA Network Open.*

≥ 1 visit: any self-harm outcome			
No	149,249 (98.0%)	292,418 (98.6%)	0.05
Yes	3,072 (2.0%)	4,064 (1.4%)	
≥ 1 visit: any psychiatric outcome (mental health, self-harm, or suicide)			
No	98,309 (64.5%)	216,151 (72.9%)	0.18
Yes	54,012 (35.5%)	80,331 (27.1%)	

Note: anxiety & neurotic disorders, adjustment reaction, behavioral disorders, mood disorders, eating disorders, other mental health, disorders of psychological development, schizophrenia, substance abuse and suicidal were used to compute the mental health outcome.

© 2022 Ledoux AA et al. *JAMA Network Open.*

eTable 3. Association Between Concussion and Outcome Measures When Adjusting for Multiple Concussions

Outcome	Hazard Ratio (95% CI)	
	**Crude[a]**	**Adjusted[b]**
Mental Health problems	1.39 (1.37, 1.40)	1.34 (1.33, 1.35)
Self-harm	1.44 (1.37, 1.51)	1.44 (1.38, 1.52)
Psychiatric hospitalization	1.43 (1.37, 1.49)	1.42 (1.36, 1.48)
Death by Suicide	1.51 (0.88, 2.58)	1.46 (0.85, 2.52)

Note: Multiple concussions was defined as emergency or primary care provider visits resulting in a concussion diagnosis at four weeks or more post-index event for cohort entry. [a]We used the matched sample to compute the crude hazard ratio. [b] Hazard ratio for a time to event analysis adjusting for residential neighborhood income quintile, child abuse or neglect, migraine history, organic mental disorders or developmental disorders, pediatric complex chronic conditions and multiple concussions.

© 2022 Ledoux AA et al. *JAMA Network Open.*

eTable 4. Association Between Multiple Concussions After the Index Visit and Study Outcome Measures

Outcome	Hazard Ratio (95% CI)
Mental Health	1.09 (1.08, 1.10)
Self-harm	1.06 (1.04, 1.07)
Psychiatric hospitalization	1.07 (1.06, 1.08)
Death by Suicide	1.09 (0.89, 1.32)

© 2022 Ledoux AA et al. *JAMA Network Open.*
